# Navigating Traditional Chinese Medicine Network Pharmacology and Computational Tools

**DOI:** 10.1155/2013/731969

**Published:** 2013-07-31

**Authors:** Ming Yang, Jia-Lei Chen, Li-Wen Xu, Guang Ji

**Affiliations:** ^1^Longhua Hospital Affiliated to Shanghai University of TCM, Shanghai 200032, China; ^2^Institute of Digestive Disease, Longhua Hospital, Shanghai University of Traditional Chinese Medicine, Shanghai 200032, China

## Abstract

The concept of “network target” has ushered in a new era in the field of traditional Chinese medicine (TCM). As a new research approach, network pharmacology is based on the analysis of network models and systems biology. Taking advantage of advancements in systems biology, a high degree of integration data analysis strategy and interpretable visualization provides deeper insights into the underlying mechanisms of TCM theories, including the principles of herb combination, biological foundations of herb or herbal formulae action, and molecular basis of TCM syndromes. In this study, we review several recent developments in TCM network pharmacology research and discuss their potential for bridging the gap between traditional and modern medicine. We briefly summarize the two main functional applications of TCM network models: understanding/uncovering and predicting/discovering. In particular, we focus on how TCM network pharmacology research is conducted and highlight different computational tools, such as network-based and machine learning algorithms, and sources that have been proposed and applied to the different steps involved in the research process. To make network pharmacology research commonplace, some basic network definitions and analysis methods are presented.

## 1. Background

Traditional Chinese medicine (TCM) has been developed and practiced in China for thousands of years. Although TCM is still being practiced and more countries consider it an alternative treatment [1], several questions need to be addressed: (1) what are the active substances in TCM and how do they work? (2) What are the combinatorial rules of TCM herbal formulae, and why can it be used for the treatment of multiple diseases? (3) What basic biological knowledge underlines TCM? The development of systems biology technology over the past several decades has helped shed light on the effectiveness of TCM and helped to answer the previous questions. Systems biology tools could be used to obtain valuable insights into TCM theories. Recent advancements in “Omics” technologies have led to more accumulated data that require powerful computational tools to study and analyze. Although the most widely used experimental technologies, such as high-throughput gene expression profiling [2], have permitted the characterization of relationships between complex biological processes and TCM treatment, an obvious limitation of these approaches is that they usually analyze data on a single state (i.e., changes in the expression of specific disease or TCM agent). To be more effective, these novel strategies should integrate systematic information to contextualize the characterization to illustrate the holistic characteristic of TCM. Such relationships could be understood better through building, validation, and analysis of computational models. Similar to complex diseases that require complex therapies, complex data require scale-matched approaches. A network-based approach for pharmacology has been proposed recently. Network pharmacology challenges the traditional “one disease-one target-one drug” paradigm and explores interactions between the body and drug by mapping drug-target-disease networks on a biological level. A recent analysis of network pharmacology highlighted the complexity of both drug action and protein-protein interaction [3–6] and triggered significant changes in strategies for therapies and the drug discovery process [7]. For TCM, Li [8] presented the framework and practice of network-based studies for understanding the mechanism of Chinese herbal formulae. His group proposed the novel concept of “network target” based on their works [9, 10], which pioneered network pharmacology research on TCM. “Network target” considers the biomolecular disease network as a target through which researchers can design and develop the best drug intervention; the key is to establish a network for drug-gene-disease association. Network pharmacology has the potential not only to accelerate TCM modernization and bridge the gap between traditional and modern medicine but also to change methods for rational design and optimization of drug discovery from herbal formulae. As a meaningful visual interface, the network-based approach is a functional element in tackling complicated problems by enabling data exploration and engaging the human ability to synthesize complex visual inputs into meaningful understanding [11]. However, the construction of a network depends on information about different types of relationships. To make network pharmacology for TCM commonplace, an all-encompassing resource that contains both TCM knowledge and biological processes and different computational algorithm tools should be refined.

In this review, we focus on how TCM network pharmacology research is conducted. We highlight different computational bioinformatics approaches that have been introduced and applied to different steps involved in network pharmacology. The general analysis process can be described as follows: (a) interaction information retrieval from databases; (b) network construction; and (c) knowledge discovery based on network models. Accordingly, this review is organized as follows: the second part contains useful databases and network analysis software. In the third part, we present the methods for constructing networks of different modes. Several basic network definitions and network properties analysis are depicted. The fourth part describes recent developments in TCM network pharmacology and discusses different computational methods that have been proposed so far to address current issues. We further discuss how and what underlying TCM knowledge can be discovered based on network models. In the final part, we discuss challenges to TCM network pharmacology. Our review is not comprehensive. Therefore, we encourage interested readers to write reviews that address another aspect of this subject.

## 2. Databases and Data Analysis Tools

**Figure 1:**
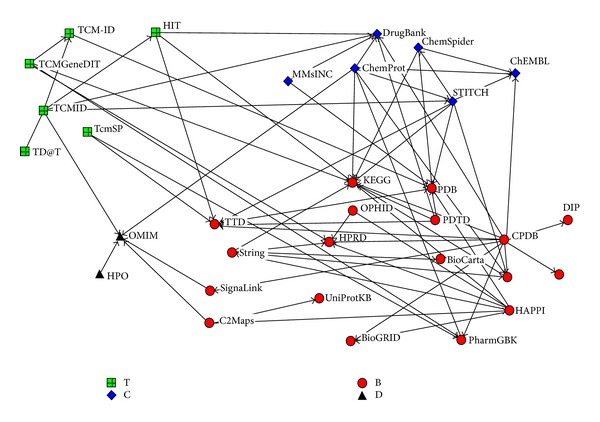
Database relationship network.

Research on network pharmacology is supported by large-scale biological databases that offer a wealth of information on interactions of biological entities, such as drug-gene-disease. These databases are developed for different but complementary objectives. With advancements in systems biology of TCM, TCM-related resources can also be obtained from the Web.  Table 1 summarizes the most frequently used resources for TCM network pharmacology. The resources are divided into the following four categories based on the type of information in the databases: (1) biomolecular databases that contain large amounts of information on human protein-protein interactions (PPIs), gene ontologies (GOs), protein-DNA interactions, and functional pathways; (2) disease/phenotype databases on phenotypes of human diseases and their related genes; (3) chemical/drug-related databases that provide many useful chemoinformatics and bioinformatics information on drugs or chemical substances, such as 2D and 3D structures, bioactivity, and comprehensive drug target (i.e., sequence, structure, and pathway); and (4) TCM-related databases that provide information on many active ingredients related to TCM drugs and their target proteins. We also focus on the relationships among these databases, mapping them in Figure 1 based on the following principle: if A database is integrated from B database or is annotated from B database, they are connected, with A having an out-degree and B having an in-degree. In other words, the database that has a larger in-degree and smaller out-degree is the source of many others and is called the primary database, whereas the database that has smaller in-degree and larger out-degree is called the secondary database.

Figure 1 shows that among the biomolecular databases, KEGG [12], HPRD [13], PDB [14], and TTD [15] have larger in-degree and smaller out-degree and could thus be considered primary databases. These databases are frequently used and provide extensive information on pathways (KEGG), PPIs (HPRD), protein structure (PDB), and therapeutic target (TTD). However, the ConsensusPathDB (CPDB) [16] and the Human Annotated and Predicted Protein Interaction (HAPPI) database [17] could be considered secondary databases. CPDB may be the largest searching platform database and integrates seven types of functional biological interactions (PPIs, signaling reactions, metabolic reactions, gene regulations, genetic interactions, drug-target interactions, and biochemical pathways) and 30 public resources. HAPPI, which integrates five different resources, was developed by Indiana University. A unified scoring model was applied to measure each PPI at one of the five-star rank levels from 1 to 5. The latest version contains 273,068, 189,150, 71,036, 33,733, and 34,770 PPIs that were ranked from 1 star to 5 stars, respectively, and provides a more flexible selection for researchers to achieve different data confidence levels. OMIM [18] under disease/phenotype databases as well as DrugBank [19] and ChEMBL [20] under chemical/drug-related databases are primary databases. TCM-related databases are all secondary databases that require integration with other resources. Although TCM has continued to gain popularity, relatively few resources on TCM are available online. These TCM-related databases complement each other to provide information on active ingredients, herbs, herbal formulae, and even herbal ingredient targets. The connection between TCM-related databases and other categories indicates to some extent the existing complex interactions of TCM-active ingredient-gene-disease. For example, TCMID [21] integrates three types of database, which is the largest data set for a related field. TCMID contains 47,000 prescriptions, 8,159 herbs, 25,210 compounds, 6,828 drugs, 3,791 diseases, and 17,521 related targets that facilitate research on TCM network pharmacology.

Table 2 lists several major network analysis tools that can be used for biological network analysis, although some were originally developed for social network analysis, such as Pajek, Ucinet, and NetMiner. Most of the tools are based on Java (Cytoscape) or Python (NetworkX, NetMiner, Guess) language script, which allows researchers to extend the functionality of network analysis by developing specific plugins or apps. More than 150 plugins are integrated in Cytoscape [22, 23], thus increasing its power and versatility. As a tool designed for biological networks, Cytoscape has several specialized plugins that can be used to import and map existing interaction data cataloged in public databases, such as BioGridPlugin [24], MiMI [25], ConsensusPathDB [26], and APID2NET [27]. Some plugins support computational literature mining. For example, AgilentLiteratureSearch [28] can mine literature abstracts from online databases such as OMIM and Medline to determine interactions. Although packages based on Matlab or R are not efficient in analyzing large networks (≥10,000 nodes), their powerful statistic and data mining toolboxes facilitate further analysis. 

## 3. Computational Measurements for Network Analysis

Network-based approaches have been proven to be helpful in organizing high-dimensional biological data sets and extracting meaningful information. The simplest way to construct a biological network is through graph points, which could be either genes, proteins, or drugs connected by lines that represent the nature of the interaction. Networks are amenable to analysis by using several branches of mathematics [29]. Thus, local and global properties of this map can be evaluated by using network metrics. In this section, we describe several of the most apparent and least complex measurements of general network analysis. Despite the simplicity of the ideas and definitions, good theoretical reasons (and some empirical evidence) support the view that these basic properties of biological networks must be very important. These measurements are discussed as follows.

### 3.1. Basic Properties

**Figure 2:**
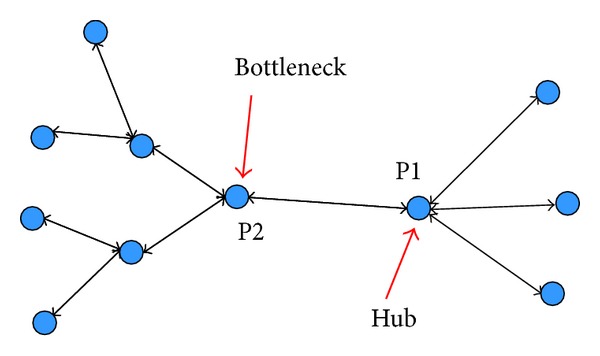
Illustrative example for measuring the basic properties of a network.

Network thinking has contributed a number of important insights on biological process. Protein and protein or disease and disease interactions are believed to be more complicated. Identifying the importance of a protein or disease is essential in understanding biological networks. The properties of the network that we are investigating primarily deal with the importance of nodes. If the group composed of important nodes is called the “center” of the biological network, we can evaluate the centrality of the nodes based on three general measurements: degree, betweenness, and closeness. Degree is the number of nodes connected to a given node in a graph. Betweenness and closeness are both related to geodesic distance, which is the number of relations in the shortest possible path from one node to another. In a biological network, a node with a large degree could be regarded as a hub node, and a node with large betweenness is a bottleneck node. Some studies suggested that human-inherited disease genes tend to be hub nodes in the interaction network [30–33]. However, other studies have provided evidence that the “hubness” of inherited disease genes may be only apparent [31] and suggested that bottleneck nodes tend to be essential proteins [34, 35]. The use of these metrics for evaluation is illustrated in Figure 2. Two proteins, P1 and P2, interact through three and two proteins, respectively. P1's proteins do not have any interactions except with P1, whereas each of P2's proteins interact with two proteins. P2 would have greater chance of influencing than P1 despite its smaller degree because it has larger betweenness, which allows greater participation in information flow and may coregulate more proteins. These network characteristics can be captured by testing the distance between two nodes. Large distances mean that diffusing information from one node to another may take a longer time or require more steps. Thus, betweenness, which is related to distance, may be more appropriate in reflecting information flow based on biological regulation [36]. However, these network centrality measurements are correlated [37] and appear to complement each other in some cases. Each of these three measurements has been elaborated in a number of ways, and the locations of nodes are described in terms of how close they are to the center of the network. Network analysts are more likely to refer to their approaches as descriptions of centrality. NIMS [38], which is a network-based approach for screening synergistic drug combinations in TCM, has integrated these three measurements into the topology score, which is used to indicate node importance. To identify genes that change their expression between two conditions, DiffRank, a novel network-based algorithm, was proposed. Betweenness was integrated into DiffRank as a structural scoring measure [39].

Thus, the distance between nodes in a network may be another important characteristic of a biological network based on the assumption that closer proteins have more similar functional annotations [40]. An analysis of network distances between regulated genes found that genes regulated by structurally similar drugs are significantly closer than genes regulated by dissimilar drugs [41]. Random walk, which describes a walker who walks randomly from node to node along edges in a network, was used to identify the relationship between disease and genes by calculating functional distance between nodes [42, 43]. The shortest path distance (SPD) is often used for network topology construction in pharmacology studies [38, 44]. For instance, SPD is applied to measure the similarity between drugs [45] or between the binding site and a ligand [46] in the context of biological interaction networks or to compare entire networks [47, 48].

### 3.2. Network Mode

**Figure 3:**
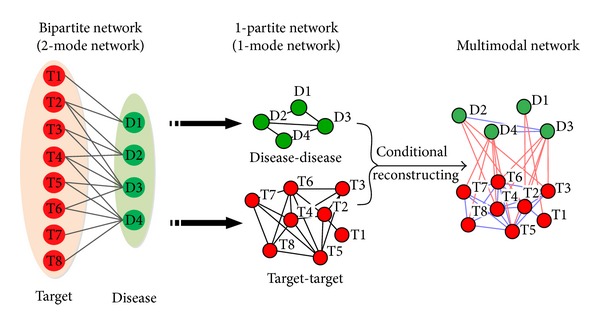
Illustrative example of network mode transformation.

A network can be classified into different modes according to the number of kinds of nodes. In general, given a network *G* = (*V*, *E*), where *V* is a set of nodes and *E* is a set of edges, if *V* has *k* subsets and no two nodes in the same subset are adjacent, *G* is called a *k*-partite network or *k*-mode network [49]. A network with two partitions is a bipartite network. A simple biological network that contains the same type of nodes such as PPIs is a 1-partite network or a standard network, where *k* equals 1. However, in many cases, biological network databases provide specialized data with different types, and researchers prefer to examine interactions between different types, such as disease-target and drug-target networks. A multi-partite network is difficult to analyze because of its asymmetry. As most network methods are developed based on the standard network, a multipartite network can be analyzed by transforming it into a single partite network, which can be easily achieved through matrix algebra.  Figure 3 shows an example of a bipartite disease-gene network. Disease nodes are D1 (its targets are T1 to T2), D2 (its targets are T2 to T4), D3 (its targets are T2 to T6), and D4 (its targets are T4 to T8). This bipartite network can be represented by matrix *M*_*ij*_, (*i* = 1,2, …, 8, and *j* = 1,2,…, 4), where *M*_*ij*_ = 1 denotes disease *D*_*j*_, which has target *T*_*i*_. This bipartite network can be transformed into two 1-mode networks through matrix multiplication after excluding self-interactions, namely, disease-disease network by *M*′∗*M* and target-target network by *M*∗*M*′. A disease-disease network is constructed by sharing a target, and a target-target network is constructed by sharing a disease. These two 1-mode networks can be analyzed by using various network methods, such as calculating basic properties. Although analyzing 1-mode networks provides deeper insights into the relationship between the same kind of entities, identifying the interactions between different entities would be more valuable. A supervised learning integration method of a bipartite network was proposed for TCM network pharmacology to identify potential targets based on known drug-protein interactions by using a predicting model [50]. The proposed approach performed better than the nearest neighbor- and weight-based algorithms. Fuzzy clustering and spectral coclustering algorithms were applied for *k*-partite network analysis in network pharmacology [49, 51]. A tripartite disease-gene-protein complex network was decomposed by using the fuzzy clustering algorithm to determine structures in a network with multiple types of nodes.

A *k*-partite network that has adjacent nodes in the same subset and is more heterogeneous, which is beyond the definition of a *k*-partite network, is called a multimodal network (MMN) [52], which is very common in biology. Metabolic pathways, gene regulation networks, and signaling pathways are some examples of MMNs whose structures are modeled heterogeneously. In the above-mentioned bipartite network transformation, two 1-mode networks are given. We reconsider the relationship between diseases based on existing information and stipulate that two diseases are correlated (nodes are adjacent) only when they share two or more targets. A simple MMN is constructed by using matrix combination, as shown in Figure 3 (rightmost portion), and reflects three kinds of relationships: disease-disease, target-target, and disease-target, which provides more information than the *k*-partite network. Complicated networks require more complicated analysis methods. CIPHER-HIT [53], a hitting time-based method that integrates modularity measure into the network inference, was proposed for the prediction of disease genes and disease subtypes on the phenotype-gene MMN. CIPHER-HIT can significantly improve disease gene predictions on modularity levels and does not require preset parameters, unlike the random walk with restart [53]. A case study on breast cancer by using CIPHER-HIT was also given in this paper; two critical breast cancer subtypes were identified, which could reveal the potential genetic and phenotypic properties of breast cancer [53]. 

### 3.3. Community Structure and Subgroup Analysis

**Figure 4:**
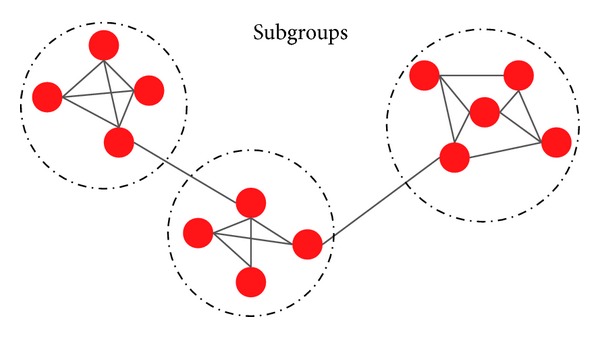
Network subgroups.

Network analysis commonly focuses on certain issues, one of which is subgroup structures.  Figure 4 illustrates a simple network with several subgroup structures in which network connections are dense, but between which they are more sparse [54]. Therefore, network structure can be viewed from three different levels: individual, subgroup, and entire network [55]. Divisions of biology entities into groups could be a very important aspect of biological network structure. In addition, analyzing the structures of PPI networks could help biologists identify important biological units such as protein complexes and functional modules [56]. Understanding how biological entities play a role in the entire network is important. For instance, in biological networks, some entities may act as bridges between subgroups and could be potential bottleneck nodes, while others may all be related within a single subgroup and could be potential hub nodes that could be important in understanding the biological process. The differences in the functions of entities may result from the different ways that entities are embedded in the structure of subgroups within a network. In TCM, most prescriptions commonly have some relatively smaller fixed composition(s) that can be called a basic formula (BF) [57]. Adding and/or subtracting herbs from BFs are usually carried out to produce a personalized treatment. Therefore, BFs could be implied by subgroups in herb-herb networks [58–60]. An herb-herb network, where subgroups overlap, provides insights into the TCM principle of treating the same disease by using different methods or treating different diseases by using the same method. Approaches to understanding the subgroup structure of a network have been developed. Some of these methods are based on graph theory, such as spectral bisection method [61] and Kernighan-Lin algorithm [62], while some are based on sociological methods, such as *k*-plexes [63], *k*-cores [64], CPM [56], and maximal clique algorithms [65–69]. Other approaches are based on clustering methods, such as optimization-related algorithms [70–73] and similarity-related methods [74, 75]. For TCM-related networks, network-based subgroup analysis methods are summarized in Table 3. Several subgroup analysis methods have been applied in the analysis of different types of networks in TCM, such as herb-herb [58], symptom-symptom [76], target-target [77], factor-factor [78], and pathway-pathway networks [79]. Thus, valuable herb combinations (basic formulae) [58], meaningful symptom groups [76], or biological entities [80] for differentiating syndromes, and instructive therapy combinations for tumor treatment [78] have been obtained and provide a more comprehensive understanding of TCM principles.

## 4. TCM Network Pharmacology

**Figure 5:**
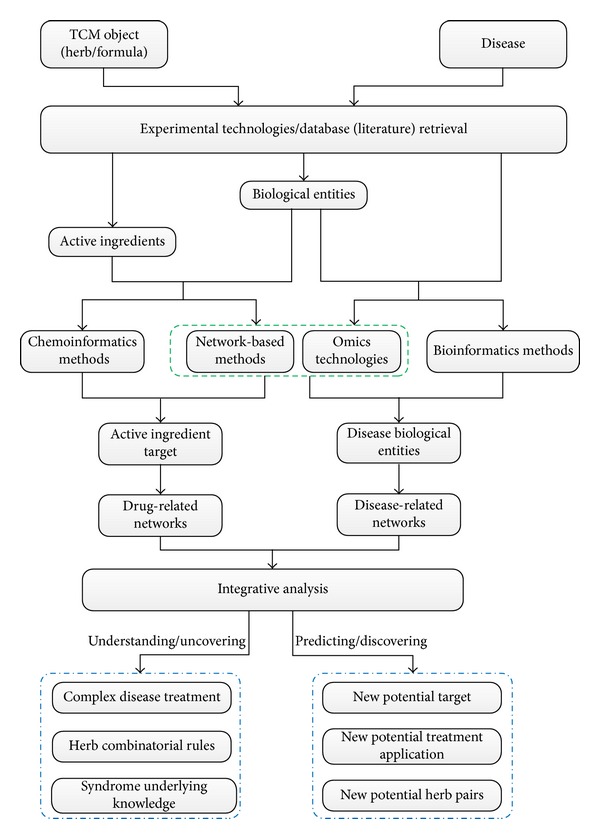
General TCM network pharmacology framework.

A general framework for TCM network pharmacology research is shown in Figure 5. The flowchart shows two main types of analysis flows, whose starting points are the TCM object and disease. The key challenge for TCM network pharmacology research is the construction of drug- and disease-related networks, which requires different steps and methods. Although high-throughput experimental technologies offer considerable information, these technologies are often expensive and time consuming. Using existing information from databases appears to be more efficient but is not consistently sufficient. Advancements in systems biology have ensured that any information on both biological/medical resources and methodology can be obtained to facilitate TCM network pharmacology research. TCM network pharmacology and modern systems biology share most resources. This resource sharing explains to some extent why researchers view TCM network pharmacology as a bridge between TCM and modern medical science. The following sections discuss the practice of TCM network pharmacology and the resources and computational methods/tools it uses.

### 4.1. Network Construction

**Figure 6:**
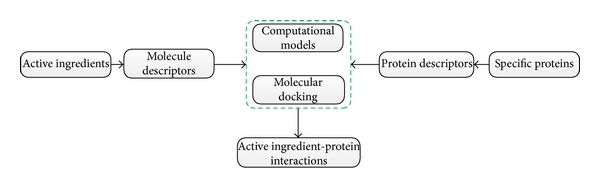
General chemoinformatics protocol for identifying AI-protein interactions.

The key in network pharmacology is the construction of the network. The network pharmacology research process usually begins from the identification of drug- or disease-related biological entities (gene, protein, and metabolite) and then proceeds by constructing drug- or disease-related networks that could reveal underlying relationships by analyzing network topology properties. However, in TCM, constructing drug-related networks is different. Drug-related networks in TCM include herb/active ingredient (AI)-herb/active ingredient (AI) network and target-target network. Herb/AI-herb/AI network can be constructed by sharing formulae, targets, or disease/phenotypes, whereas target-target network can be constructed by sharing herb/AI. As herbal formulae are a major form for treatment in TCM, herb-herb networks that could reflect herbal combinatorial rules are particularly interesting. Li et al. [59] initiated a TCM network pharmacology based on an herb-herb network and proposed a DMIM method for constructing the network that assessed the herb-herb relationship based on both distance score and mutation information association. Identification of AI in herbs is the first step. TCM modernization in the past few decades has enabled the retrieval of most known AIs in herbs from the literature or databases, although available data are not comprehensive. The challenge is to detect AI targets. AI identification is an easy method for constructing TCM drug-related networks based on existing resources. However, a shortcoming of current TCM-related interaction databases is that they contain a rather small number of interactions that have been validated experimentally. Many interactions remain unknown. Thus, methods that predict and identify new interactions should be developed. Experimental technologies such as “Omics” technologies are beyond the scope of this review.  Table 4 summarizes useful methods or algorithms for AI target identification. Although only a few of these methods have been applied for TCM, all are instructive and could facilitate TCM drug-related network construction. Figure 5 shows the two main computational strategies for AI target identification: chemoinformatics and network-based methods. The goal of chemoinformatics is to describe relationships between targets and ligand- or structure-based information from AI. The general protocol of chemoinformatics for identifying AI-protein interactions is shown in Figure 6. First, structure information of AI is retrieved from databases such as ChEMBL (https://www.ebi.ac.uk/chembl) or TCM Database@Taiwan (http://tcm.cmu.edu.tw). The structure information is then imported into a chemical software such as Dragon (Talete Inc.), Cerius2 (Accelrys, Inc.), MOE (Chemical Computing Group Inc.), or Sybyl (Tripos Inc.) to calculate the molecular descriptors, while protein descriptors are obtained from databases such as PDB (http://www.rcsb.org/pdb). Second, molecular docking is performed to infer the relationship between ligand and protein, or computational models are established to model the relationship between molecular and protein descriptors. Finally, AI-protein interactions are obtained through model prediction or by ranking the dock score. In this strategy, supervised machine learning algorithms such as k-nearest neighbors (KNNs) [81], support vector machine (SVM) [82–84], random forests (RFs) [82, 83], and Bayesian classifiers [85–87] are often used to establish classification models (Table 4). These algorithms require known AI-target information that is usually obtained from DrugBank (http://www.drugbank.ca) to train the models to successfully predict unknown information. Li et al. [83] proposed a network-based approach to reveal the mechanisms of action of three representative Chinese herbs (Ligusticum chuanxiong Hort., Dalbergia odorifera T. Chen, and Corydalis yanhusuo WT Wang) that are used to treat cardiovascular disease (CVD). RF and SVM were used to establish the drug-target models based on 6,511 drugs and 3,999 targets extracted from DrugBank databases, which indicates good prediction performance for drug-target interactions [82]. The AI molecular descriptors of the herbs were then calculated by using Dragon, and the structure information of candidate proteins was retrieved from the PDB database. As a result, 261 protein targets related to 64 AIs were predicted for the construction of a drug-target network. In this study, SVM was also used to predict oral bioavailability (OB) for screening AIs such that only AIs with good OB were selected for further analysis. KNN was applied to predict drug-target interactions [81]. In this paper, the common functional groups of drugs, instead of molecular descriptors, and four functional groups of proteins (enzymes, ion channels, G-protein-coupled receptors, and nuclear receptors), instead of an entire family, were used to establish the classification models. In some cases, unsupervised algorithms such as self-organizing maps are useful [88, 89] and can be used to assess similarities between chemical and protein features. However, most chemoinformatic methods often focus on a handful of proteins without considering that similar drug responses may result from their different targets in the same pathway or in the same biological process rather than from having common targets [87, 90, 91]. Information on drugs of one target and its distance in biological space to other targets can support the evaluation of new molecules for one or more novel targets [92]. Recent studies that combine different types of data such as protein-protein interactions have shown how computational analysis can identify drug targets [91, 93, 94]. Network-based approaches such as drugCIPHER [91] and WNBI [93] are good examples (Table 4). DrugCIPHER [91] integrates both drug therapeutic similarity (TS) and chemical similarity (CS) and uses a network topology property, namely, drug-protein closeness based on the PPI network, as drug genomic relatedness to model the relationships between drugs and targets. Three linear regression models, namely, drugCIPHER-TS, drugCIPHER-CS, and drugCIPHER-MS, which relate TS, CS, and their combination, respectively, are established. A comparison indicates that drugCIPHER-MS performs significantly better than the others, having successfully predicted the high-ranking proteins of Oxytocin and Nefazodone in the database. WNBI [93] integrated both drug-based and target-based similarity inference. Node-weighted network-based inference and edge-weighted network-based inference are then proposed by matrix operation. This approach can handle the weighted drug-target interaction network.

Constructing disease-related networks is not easy because disease biology is extremely complex. The progress of high-throughput interaction discovery experimental technologies enhanced the quality of PPI maps, which have become valuable tools that help in understanding the underlying mechanisms of diseases [95]. A prerequisite to the construction of disease networks is the availability of interaction information. Disease-related networks include disease-gene/protein, gene/protein-gene/protein, disease-phenotype, phenotype-phenotype, and disease-disease networks, among others. A critical step for the construction of disease-related networks is the identification of disease-gene/protein interactions. These interactions can be achieved in various ways, which is similar to the identification of AI targets. Most TCM network pharmacology researchers retrieve disease gene/protein based on databases such as OMIM (http://www.omim.org), whereas others develop computational methods to assess the susceptibility of genes to diseases. Some of these methods are based on bioinformatics models such as machine learning algorithms (Table 4). These methods, which are mostly based on gene expression pattern recognition, assume that some disease genes are already known and detect candidate genes based on established classification models; SVM [96, 97] and Bayesian classifiers [98, 99] are often used. Microarray gene expression data sets contain a large number of features. Thus, several dimension reduction methods are useful, such as principal component analysis [100] and maximum relevance minimum redundancy [81]. Other methods are network-based approaches (Table 4) that integrate different types of data for analysis. Wu et al. [101] developed the network-based tool CIPHER to predict disease genes. CIPHER was based on the characteristics of genes that share a mutant phenotype, which are closely linked in the network. This approach integrates phenotype similarity and gene closeness based on the PPI network and uses their correlation as a disease predictor to establish the linear model. LMMA [102] was proposed by the same group and was developed for disease-related network construction, which combined text mining and multivariate statistics. LMMA initially constructs a literature mining-based network (LM) by using literature information from a database such as PubMed (http://www.ncbi.nlm.nih.gov/pubmed). The microarray information is then integrated into the approach. The construction of an LMMA-based network is facilitated after the LM-based network was refined through stepwise multiple variable selection. LMMA was applied for the construction of angiogenesis network. Compared with the LM-based approach, LMMA could significantly eliminate false positive relations to obtain a more reliable interaction network. Some recent subgroup analysis-based approaches, such as MIClique [103], WSM [104], and CPM [105], can identify the disease-gene relationship. Most of these approaches were not applied on TCM network pharmacology, but they are all instructive.

Similarity ensemble method (SEM) is widely used in many aspects of network pharmacology research. In contrast to model-based approaches, SEM offers a model-free alternative because of its nonparametric characteristics [106]. Similarity metric, Jaccard similarity coefficient [107], and Tanimoto similarity coefficient [108, 109] are often used in TCM network pharmacology research to assess GO function similarity [38], compound structure similarity [110], or drug-likeness calculation [111]. These methods are adopted because network pharmacology variables are usually binary coded strings. These methods originated from different cases, but they are mathematically equal [112].

### 4.2. Integrative Analysis

Integrative analysis is a complicated process in network pharmacology research. Researchers are now compelled to handle different types of lines and nodes because of multiple network construction. An easy solution is the use of functional annotation analysis for common elements based on prior knowledge. For example, 54 targets and 9 signal pathways were extracted from a CVD-related gene network after TCM drug-target network construction. These targets could reveal the biological mechanism of herbs used for treating CVD [83]. A comparison among network topology properties is also useful. Ye et al. [113] investigated the mechanism of Chuanxiong Rhizome-Paeonia Albifora Pall (HP CXR-PAP) in osteoarthritis treatment. Some similar characteristic distributions of network properties between herbal ligand-target network and drug-ligand network (data source from DrugBank) indicate that the mechanism of HP CXR-PAP on this disease has potential drug-likeness or lead-like compounds. Li et al. [59] compared the average shortest path distance (ASPD) between networks and found that ASPD between TCM drug-target and specific disease genes was significantly smaller compared with that between TCM drug-target and a randomly selected disease. This finding implies the rationality of these TCM drugs in treating specific diseases. Some useful alignment methods such as IsoRankN [114] can also identify the best mapping based on clustering and allows multiple network comparison. Module approaches for integrative analysis are more informative. Searching for modules is relatively easy if the network is simple. Thus, several network-based subgroup methods (Table 3) can be used. However, biological networks in most cases are composed of multiple types of nodes and edges. The “comodule” approach, which is another effective computational analysis method, was initially proposed by Kutalik et al. [115] and introduced to TCM pharmacology by Li et al. [59, 116, 117]; “comodule” does not have a precise definition. This approach is an analysis strategy rather than a tool in network pharmacology. The basic idea of comodule is to search modules (subsets) in heterogeneous (multimodal) or multilevel networks with similar patterns and perform an integrative analysis of their connections between or within groups. Li et al.[59] initially performed comodule analysis on multilevel networks to determine the combination rule of TCM formula. The herb, biomolecular, and disease modules in this module were extracted from herb, biological, and disease networks, respectively. Biomolecular modules support the treatment of specific disease modules by herb modules through overlapping and functional annotation analysis. Comodule analysis also allows the investigation of multiple types of lines and nodes.  Table 4 lists a number of comodule methods. ISA [118], PPA [115], and CIPHER-HIT [53] can handle two distinct types of node associations and their shared node modules on the network. comCIPHER can detect modules on a network that has three types of node relationships, such as the drug-gene-disease heterogeneous network. This method handles the dataset as a huge matrix. Row denotes gene space, whereas column denotes drug and disease spaces. Markov chain Monte Carlo was used to initially select genes as modules in the row space. The chain determined by using the Gibbs sampler and the Metropolis-Hastings algorithm is then moved. Partitioning was performed in the row and the column spaces to divide the genes into different modules. The column space (drug and disease) was partitioned into two categories, namely, associated and nonassociated with the same row of gene module. Comodules, including genes and their drugs and diseases, could be achieved through Bayesian partitioning after presenting the distributions of drug-gene and disease-gene profile values. This algorithm has two advantages. First, the drug-gene and disease-gene relationships are simultaneously investigated within the same module to facilitate the identification of potential associations between drugs and diseases. These associations are meaningful and might suggest new drug applications and side effects. This paper shows that comCIPHER successfully identified two drugs (Pranlukast and Minocycline) as new treatment for human cancer. Second, modules obtained by using comCIPHER seem more compressed compared with those obtained by using other module analysis methods such as PPA. This finding might provide a clearer insight into the association between drugs and diseases because of high network interconnections.

### 4.3. Applications

Network pharmacology, as a distinctive new approach for TCM research, includes the application of network analysis to identify the group of proteins that are most critical in diseases and to recognize chemical molecules that can target that group of proteins. Network pharmacology is similar to other computational tools and generally has two main functions (Figure 5). One function is the understanding/uncovering function, which involves providing a deeper insight or scientific evidence for TCM knowledge or breaking down existing TCM knowledge and identifying them as scientifically proven. The other function is the predicting/discovering function, which involves extending knowledge or providing new hypotheses by building on existing TCM knowledge by using more reliable network models. The following sections discuss these functions in detail.

#### 4.3.1. Understanding/Uncovering the TCM Principle of Treating Complex Diseases

TCM treatments are holistic, considering the patient as a whole rather than focusing solely on the disease. This characteristic agrees with the concept that various complex diseases result from dysregulation of multiple pathways and changes in expression of a large number of genes, proteins, and metabolites. Network pharmacology provides a deeper insight into TCM treatments and helps uncover action mechanisms on a biological basis. Recent progress in TCM network pharmacology research revealed the biological molecular mechanisms of TCM treatment of many complex diseases (Table 5). CVD is a class of diseases that involves dysfunction of the heart or blood vessels. Zhao et al. [119] identified 1,619 proteins involved in 33 pathways after mapping CVD drug targets from DrugBank. These proteins could be regarded as candidate protein targets related to CVD. Different medications are employed to treat this disease. TCM herbs or formulae that can effectively promote blood circulation for removing blood stasis (“*Huo Xue Hua Yu*”), such as *Salvia Miltiorrhiza*, *Ligustici Chuanxiong*, and *Panax Notoginseng*, are often used. Li et al. [37] constructed a compound-potential target network and a compound-pathway network based on the *Compound Danshen Formula *(CDF). This approach identified 41 potential targets of CDF that are significantly related to CVD and the involvement of three main pathways, namely, PPAR signaling, glucocorticoid and inflammatory, and L-arginine/NO signaling pathways. Wang et al. [120] proposed the network pharmacology method to investigate the mechanisms of four clinically and widely used herbs (*Radix Astragali Mongolici, Radix Puerariae Lobatae, Radix Ophiopogonis Japonici, *and* Radix Salviae Miltiorrhiza*) for CVD treatment. Twenty-one out of 68, 19 out of 77, 13 out of 34, and 19 out of 77 targets were related to CVD, respectively. Astragaloside IV, one of the main AIs of *Astragalus Membranaceus*, identified 39 distinct proteins as putative targets related to CVD. Thirty-three proteins can be classified into eight functional classes that are related with CVD pathogenesis, such as the regulation of vasoconstriction and vasodilation, blood coagulation, calcium ion related, MAP kinase activity related, and others [119]. Rheumatoid arthritis (RA) is induced by several complex processes, including inflammatory response, excess synovial fluid, and the development of fibrous tissue in the synovium [121]. TCM regards RA as a blockage disease. Thus, the main treatment principle for RA is the removal of dampness and dredging the channel [122]. Several studies in network pharmacology [77, 123, 124] provided biological molecule evidence for the rationality of this principle. Wu-Tou-Tang (WTT) [77] and Qin-Luo-Yin (QLY) [124] are classical TCM formulae that could be used for treating RA. WTT [77] is composed of five herbs, namely, *Radix Aconiti, Herba Ephedrae, Radix Astragali, Raidix Paeoniae Alba, *and* Radix Glycyrrhizae*. Yan et al. [77] collected the structure information of 165 compounds of WTT. After analyzing the topological features of both PPI and drug-target networks, nine proteins with higher values of centrality properties were identified as major candidates of effector modules of WTT. Six proteins, namely, ADRB2, ADRA1B, HSP90AA1, STAT3, NR3C1, and TUBB, were significantly associated with RA. Twelve proteins/genes in QLY are related with RA. These proteins were related to angiogenesis, inflammatory response, immune response, and NF-*κ*B activity.

#### 4.3.2. Understanding/Uncovering Herb Combinatorial Rules in TCM

The role of herbs in TCM formulae should be understood because their combinatorial rules might reflect underlying principles of TCM therapies. TCM formulae are composed of herbs that play different roles during treatment. “Jun” represents the principal component and treats the main disease directly. Other herbs, namely, “Chen” (minister), “Zuo” (adjuvant), and “Shi” (courier) [125], help enhance the effects, treat the accompanying symptoms, and facilitate the delivery of the principal component, respectively. Several researchers [37, 111, 124] provided some good examples to clarify the roles of herbs in formulae at a biological molecular level by using network pharmacology. Zhang et al. [124] examined the roles of herbs in QLY for RA treatment. QLY is composed of four herbs, namely, *Sophora Flavescens *(SF),* Sinomenium Acutum *(SA),* Phellodendron Chinensis* (PC), and* Dioscorea Collettii *(DC). Target network analysis and functional annotation analysis indicate that SF, which is a “Jun” herb, performs principal processes in the development of RA. These processes include angiogenesis, inflammatory response, and immune response, which are consistent with the function of this herb. Other herbs served as complements by regulating RA-related genes. Other studies [37, 111] examined herb combinatorial rules based on OB prediction before network construction. Tao et al. [111] explained the combinatorial mechanism of *Radix Curcumae* formula (RCF) and predicted the potential targets related to CVD. RCF includes four herbs, namely, *Radix Curcumae* (RC), *Fructus Gardeniae* (FG), *Moschus* (MS), and *Borneolum *(BM). This paper predicted the OB of herbal ingredients based on the developed silicomodel [126]. Drug-likeness index was calculated based on Tanimoto similarity. OB and drug-likeness were used to select candidate compounds. Seventy-four candidate compounds with good OB were obtained. The number of candidate compounds explained the roles of herbs in this formula. Forty-five out of 74 compounds were involved in the “Jun” herb (RC), 19 out of 74 compounds were involved in the “Chen” herb (FG), 12 compounds were involved in the “Zuo” herb (MS), and only three compounds were involved in the “Shi” herb (BM). The percentage of overlapping targets also supported the combinatorial rule. The number of shared targets between “Jun” and “Chen” was larger than that between “Jun” and “Zuo.” No shared targets were found among “Jun,” “Chen,” and “Shi.” These results illustrate the different roles of herbs in RCF for CVD treatment. Their study investigated the mechanisms of CDF for the same disease. The results also indicate the feasibility of this analysis to uncover the herb combinatorial rules in TCM formulae [37].

#### 4.3.3. Understanding/Uncovering the Underlying Principle of TCM Syndromes

Syndrome is the basic concept in TCM theory. Most of its contents are abstracted and inferred from direct observation and experience. Syndrome differentiation guides TCM therapies. Given the importance of syndrome differentiation, its underlying principle should therefore be investigated. Network pharmacology is a powerful tool for understanding TCM syndrome on a molecular level [10, 127]. Li et al. pioneered this approach [10, 127, 128] and explored relationships between syndrome-related diseases and the neuroendocrine-immune (NEI) system based on the basic properties of a syndrome network (hot and cold) [127, 128]. A hot syndrome network was constructed based on 38 related diseases, and a cold syndrome network was constructed based on 21 related diseases. Biological entities as network nodes were classified into hot and cold genes based on a predefined topological temperature. The study conducted functional annotation analysis for hub nodes of networks and topological temperature comparison, which indicated that the molecular foundation of hot syndrome was mainly associated with immune-related genes, and cold syndrome was primarily based on hormone-related genes [127]. Ma et al. [128] selected 16 family members that have a history of cold syndrome to examine gene expression levels. Twenty-five differentially expressed genes were identified. Thirteen genes interacted with NEI cold or hot genes by expanding the network based on PPIs. Twelve pathways of these interaction genes were identified as metabolism- or energy-related, which indicated the relationship between TCM syndrome and energy metabolism in the context of the NEI network. The natural properties of herbs may indicate the principle behind TCM, such as “cooling the hot and warming the cold.” Two classical formulae, namely, CWHF and HCHF, were applied to the rat model of collagen-induced arthritis after identifying the hub genes of the cold and hot networks. These formulae represent cold syndrome-oriented and hot syndrome-oriented herbal treatments, respectively. CWHF suppresses the hub genes of the cold network, and HCHF tends to affect the hub genes of the hot network [127]. In another study, Li et al. [59] found that major ingredients paired with “warm” herbs caused synergistic proangiogenic activity. Their recent study [129] further explored hot and cold syndromes by using a network balance model. Bioinformatics and clinical information were combined to establish the network model for identifying biomarkers that reflect network imbalance in hot/cold syndromes to reveal the biological basis of cold and hot syndromes in chronic gastritis patients. Thus, several biomarkers were identified. Higher leptin levels were found in cold syndrome patients, whereas higher CCL2/MCP1 levels were found in hot syndrome patients. These findings further revealed the connections between TCM syndromes and the metabolism and immune system. The potential of tongue-coating microbiome as a biomarker for characterizing TCM syndromes was also discussed [130]. Tongue-coating samples were collected from 19 gastritis patients and 8 healthy volunteers. These patients were categorized into hot and cold syndromes based on traditional tongue diagnosis. Next-generation sequencing data analysis indicated that a total of 381 species-level operational taxonomic units (OTUs) differed significantly between groups. Two hundred fifty-one of these OTUs were classified into 61 genera and 49 species. These genera and species could be regarded as potential biomarkers for characterizing hot/cold syndromes.

Lu et al. explored the molecular mechanism of TCM syndrome on RA patients through network pharmacology [80, 117, 123, 131]. Their findings indicate that the cold and hot syndromes of RA patients can be differentiated based on biological modules. Thirty-three RA patients with cold and hot syndromes were included. Twenty-one significantly differentially expressed genes were identified between cold and hot syndromes after genome-wide expression analysis. RA-related network was constructed by expending the PPI network by using these genes as seeds. Four significantly and highly connected groups were obtained after subgroup network analysis. Group 1 was mostly associated with signal transduction. Group 2 was related to eicosanoid metabolic processes, oxidation-reduction reactions, and fatty acid metabolic processes. Groups 3 and 4 were involved in cell proliferation [80]. Their other study [131] included healthy volunteers to further explore the difference of biological basis of TCM syndrome between RA and normal patients. Thirty-five differentially expressed genes were identified between the cold syndrome and normal patients, and 21 genes were identified between hot syndrome and normal patients by using similar strategy analysis. Their shared genes were related to the following pathways: autoimmune thyroid disease, cell adhesion molecules, T-cell receptor signaling pathway, rheumatoid arthritis, and proteasome. These pathways also indicated the different molecular basis between RA and the normal patients. Jiang et al. then investigated the mechanism of effect of TCM syndrome on the clinical effectiveness of interventions [123]. Different therapies showed different benefits in treating RA patients with different TCM syndromes. For example, TCM therapy is more appropriate for hot syndrome, whereas biomedical therapy is better for cold syndrome. These results clarify the relationship between biological modules and TCM syndromes.

#### 4.3.4. Predicting/Discovering New Potential Targets and Treatment Applications

The predicting/discovering function of network pharmacology as a computational tool is mainly based on the assumption that other nodes, which are topologically closely related to them or their neighbors, might also be associated if significant node pairs in the network are known to be associated. The association is not guaranteed, but it can be used to facilitate the direction of laboratory testing or to validate and lead to new discoveries. The predicting/discovering function of new potential targets of drug is valuable for providing new insights into the mechanism of drug action and might lead to new treatment applications. Zhang et al. [132] applied TCM network pharmacology to explore vitexicarpin (VIT). VIT is extracted from the fruits of *Vitex rotundifolia*. They [59] previously found that VIT has antiangiogenic properties, but the mechanism remains unknown. This study used drugCIPHER [91] to predict the target proteins of VIT. The top 10% targets of VIT predicted by drugCIPHER model were selected to construct a drug-target network to identify significant pathways. Fifty-eight targets of FDA-approved drugs that directly targeted VEGF signaling pathways were also collected. Eleven direct target proteins were obtained based on correlations between the profiles of the 58 FDA-approved drugs and VIT. SRC and AKT, whose drugCIPHER scores are ranked at the top 2, were validated by experiments and computational docking analysis. Thus, the potential targets of new VIT predicted by network-based approach illustrate the mechanism of its antiangiogenic activity and lead to its new application as an angiogenesis inhibitor. Another article provides new insights into rhein [133], which is a classical natural substance isolated from rhubarb. This study successfully predicted three new molecular targets for rhein, namely, MMP2, MMP9, and TNF. MMP2 and MMP9 were significantly associated with cancer-related pathways, which further illustrates the potential of rhein and its products to be used for cancer relief in China. Gu et al. [134] conducted network analysis to elucidate the action mechanism of the medical composition, Tangminling Pills (TP). TP was designed for the treatment of type II diabetes mellitus (T2DM). A total of 676 ingredients contained in TP were considered for the construction of drug-target and drug-drug networks. Five ingredients were significantly associated with T2DM through subgroup and topology property analysis, namely, rheidin A, rheidin C, sennoside C, procyanidin C1, and dihydrobaicalin. Their biological activities of T2DM were not reported. These findings might expand the applications of these ingredients. A drug-target network of Yuanhu-Zhitong (YZP) was constructed to explain its molecular mechanism [135]. YZP is a classical formula in TCM and is widely used for the treatment of gastralgia, dysmenorrhea, and headache. The alkaloids of YZP are highly connected with the GABA receptor group, which are close to benzodiazepine receptors. This finding suggests that YZP might serve as an antidepressant and an antianxiety drug. These potential treatment applications were validated by computational docking analysis and experiments. Some new indications of CDF (a classical TCM formula) were also reported [37]. Li et al. [37] found that CDF may be potentially applied to treat metabolic diseases because of its high association with metabolism-related targets after network pharmacology analysis. These findings may drive future laboratory or clinical research. However, they have not been further validated.

#### 4.3.5. Predicting/Discovering New Potential Synergistic Herb/Ingredient Pairs

An herb pair, which is the most frequent cooccurrence of two herbs in TCM therapies, is the basic herbal combinatorial form in TCM formulae. Herb pairs may achieve better efficacy according to TCM theory. Hundreds of herb pairs are available in TCM therapies, but their function in the treatment remains unknown. Discovering new potential synergistic herb/ingredient pairs is important for understanding combinatorial rules and designing new TCM drug compositions. Herb pairs can be mathematically denoted as the interaction between two herbs. Edges in the network depict this relationship. Network-based approaches were proposed to explore the relationship of herbs to achieve core herbs, core herb pairs, and core herb formulae [58, 59, 136–138]. Li et al. [59] discovered six new herb pairs related to angiogenic activities by DMIM based on an herb-herb network. Three of these pairs included *Rhizoma Chuanxiong* (RCX), which indicate the importance of this herb. Further network topology analysis also supported the role of RCX as a core herb. This herb-herb network also successfully retrieved most widely known herb pairs and six classical herbal formulae, which indicate its reliability to a certain extent. A new herb pair, RCX and *Flos Carthami* (FC), was chosen to evaluate the combination effect. This work utilized tetramethylpyrazine, a compound isolated from RCX, instead of RCX and hydroxysafflor yellow A, a compound isolated from FC. The results validated the synergistic effect of this herb pair, which also expanded their applications in clinical therapies in TCM. Their study [38] explored 63 agents, including 61 herbs or herb ingredients and their combination effect related to antiangiogenesis by using NIMS. The advantage of NIMS is its ability to integrate two informative parameters, topology score, and agent score, which might increase the reliability and robustness of outputs. Thus, five new synergistic herbal ingredient pairs were reported, which were experimentally validated. The rank order of maximum increased inhibition rate of ingredient pairs obtained from experiments was identical to that predicted by NIMS, which further confirmed the synergistic effect of these ingredient pairs.

## 5. Perspectives

An overview of TCM network pharmacology and its computational tools was presented. Network pharmacology, as a new research approach, provides revolutionary opportunities for TCM modernization. Recent studies show that sufficient information can be obtained to largely enhance understanding of the underlying principle of TCM when combined with multiple types of data and computational tools. It might predict and explain existing TCM knowledge. Recent successes in TCM network pharmacology research were achieved in the last decade. However, current TCM network pharmacology remains in its infancy, and deducing reliable predictive inferences remains challenging because of a number of reasons. First, network pharmacology largely relies on available data sources. Several biological databases are open source and up to date. Thus, more information on TCM is needed, including the standardization and identification of active ingredients, which requires additional experimental technologies and further experimental investigations into TCM-related biochemistry research to better understand the mechanisms of TCM drug action. Second, this information is collected from various experiments or literature, thereby resulting in many false positive and false negative interactions that can be partially attributed to the lack of reliability and robustness of network models. Therefore, more powerful computational tools are needed to reevaluate or to refine more informative interactions. Third, network-based algorithms have advantages for the analysis of multiple types of data. However, several current informative network-based algorithms are limited by network scale because of their computational cost. Most algorithms are designed for the analysis of a static network, which ignores the dynamic nature of molecular systems. Thus, high-performance computational tools for analyzing large-scale networks and dynamic networks should be developed for rapid and efficient analysis. Lastly, the results of network pharmacology studies should be validated to verify the inferences. The associations, especially for TCM ingredients and their interactions, may not be strong enough to be easily identified by general experiments. Thus, more sensitive and quantitative experimental techniques are needed. Most TCM network pharmacology studies focus on the efficacy of an herb or formulae. However, concerns over drug toxicity increased significantly in the past decade. Research on the mechanism of adverse side effects or identification of the “off-targets” of TCM drug is valuable for the reevaluation of TCM clinical efficacy and the design of new TCM therapies, which may become the future direction of TCM network pharmacology research. Another interesting aspect is the interactions between TCM and Western medicines, which may illustrate how the combination can achieve better efficacy and fewer side effects. The use of network pharmacology approaches is vital to driving future research on TCM pharmacology.

## Figures and Tables

**Table 1:** Useful public databases for TCM network pharmacology.

Type^#^	Name	Description	Application	Webpage	Reference
B	OPHID	Online predicted human interaction database: a web-based database of predicted interactions between human proteins, which contains 23889 predicted interactions currently	PPIs retrieval	http://ophid.utoronto.ca	[139]
	STRING	A database of known and predicted protein interactions	PPIs retrieval	http://string-db.org/	[140]
	BioGRID	Biological general repository for interaction datasets: providing protein-protein interaction data from model organisms and humans	PPIs retrieval	http://thebiogrid.org/	[24]
	HPRD	Human protein reference database: depicting and integrating information related to domain architecture, posttranslational modifications, interaction networks, and disease association for each protein in the human proteome	PPIs retrieval	http://www.hprd.org/	[13]
	HAPPI	Human annotated and predicted protein interaction database: containing 142,956 nonredundant, medium to high-confidence level human protein interaction pairs among 10,592 human proteins	PPIs retrieval	http://bio.informatics.iupui.edu/HAPPI/	[17]
	PDB	Protein data bank: a key resource in areas of structural genomics for containing 3D biological macromolecular structure	Protein information retrieval	http://www.rcsb.org/pdb/	[14]
	PDTD	PDTD: a web-accessible protein database for drug target identification and focusing on those drug targets with known 3D structures	Drug target identification	http://www.dddc.ac.cn/pdtd/	[141]
	TTD	Therapeutic target database: providing information about the known and exploring therapeutic protein and nucleic acid targets, the targeted disease, pathway information, and the corresponding drugs	Drug target identification	http://bidd.nus.edu.sg/group/cjttd/	[15]
	UniProtKB	Universal protein knowledge database: providing protein information in detail	Protein analysis	http://www.uniprot.org/uniprot/	[142]
	PharmGBK	Pharmacogenomics knowledge base: providing information of gene-drug associations and genotype-phenotype relationships	Comprehensive gene-drug-phenotype analysis	http://www.pharmgkb.org/	[143]
	DIP	Database of interacting proteins	PPIs analysis	http://dip.doe-mbi.ucla.edu	[144]
	C2Maps	A network pharmacology database with comprehensive disease-gene-drug connectivity relationships	Comprehensive gene-drug-disease analysis	http://bio.informatics.iupui.edu/	[145]
	MetaCore	An integrated suite for functional analysis of microarray, metabolic, SAGE, proteomics, siRNA, microRNA, and screening data	Comprehensive biological analysis	http://www.genego.com	[146, 147]
	CPDB	A database that integrates different types of functional interactions including protein-protein, genetic, metabolic, signaling, gene regulatory, and drug-target interactions	Comprehensive gene-drug-disease analysis	http://cpdb.molgen.mpg.de/	[16]
	BioCarta	An interactive web-based resource giving four categories information: gene function, proteomic pathways, and research reagents	PPIs and pathway retrieval	http://www.biocarta.com/	[148]
	KEGG	As a collection of online databases, which deals with genomes, enzymatic pathways, and biological chemicals, especially giving pathway map in the forms of molecular networks	PPIs and pathway retrieval	http://www.genome.jp/kegg/	[12]
	SignaLink	A database containing eight major signaling pathways, which can be used for comparative and cross-talk analyses of signaling pathways	Pathway analysis	http://signalink.org/	[149]
	Reactome	Curated knowledge base of biological pathways in humans	Pathway analysis	http://www.reactome.org	[150]
	NetPath	A manually curated resource of signal transduction pathways in humans	Pathway analysis	http://www.netpath.org/	[151]

D	OMIM	Database of comprehensive, authoritative compendium of human genes and genetic phenotypes	Disease-gene retrieval	http://www.omim.org/	[18]
	COSMIC	A database of catalogue of somatic mutations in cancer	Biological information relating to human cancers retrieval	http://cancer.sanger.ac.uk/cancergenome/projects/cosmic/	[152]
	HPO	Human phenotype ontology database: providing a standardized vocabulary of phenotype of human disease	Phenotype retrieval	http://www.human-phenotype-ontology.org/	[153]

C	STITCH	Chemical-protein interactions database: providing known and predicted interactions of chemicals and proteins	Chemical-protein interaction retrieval	http://stitch.embl.de/	[154]
	DrugBank	A knowledge base for drugs, drug actions, and drug targets	Comprehensive analysis for approved drugs	http://www.drugbank.ca/	[19]
	ChEMBL	A database of bioactive drug-like small molecules, which contains 2 D structures, calculated properties, and abstracted bioactivities	Ingredient and drug chemoinformatics information retrieval	https://www.ebi.ac.uk/chembl/	[20]
	MMsINC	A large-scale chemoinformatics database	Ingredient and drug chemoinformatics information retrieval	http://mms.dsfarm.unipd.it/MMsINC/search/	[155]
	CB	A comprehensive chemical structures database	Ingredient and drug chemoinformatics information retrieval	http://www.chemicalbook.com/	[156]
	ChemProt	A comprehensive disease-chemical biology database	Chemical-protein interaction analysis	http://www.cbs.dtu.dk/services/ChemProt-2.0/	[157]
	LookChem	A comprehensive chemical structures database	Ingredient and drug chemoinformatics information retrieval	http://www.lookchem.com/	[158]
	ChemSpider	A chemical structure database providing structures, properties, and associated information of compound	Ingredient and drug chemoinformatics information retrieval	http://www.chemspider.com/	[159]

T	HIT	A comprehensive and fully curated database for linking herbal active ingredients to targets	Herbal ingredients' targets identification	http://lifecenter.sgst.cn/hit/	[160]
	CHMIS-C	A comprehensive herbal medicine information system for cancer	Comprehensive analysis for ingredient target of cancer	http://sw16.im.med.umich.edu/chmis-c/	[161]
	TD@T	TCM Database@Taiwan: providing chemical composition of Chinese medicinal herb including two- and three-dimensional structures of each TCM constituent	TCM medical compound retrieval	http://tcm.cmu.edu.tw/	[162]
	TCMGeneDIT	A database for associated traditional Chinese medicine, gene and disease information using text mining	Comprehensive analysis for ingredient-gene disease-effect of TCM	http://tcm.lifescience.ntu.edu.tw/	[163]
	TCM-ID	Traditional Chinese medicine information database: providing information on formulae, medicinal herbs, and herbal ingredients	TCM formula and medical compound retrieval	http://tcm.cz3.nus.edu.sg/group/tcm-id/tcmid_ns.asp	[164]
	TCMID	Traditional Chinese medicine integrated database: a comprehensive database to provide information on drug-herb and its ingredient, prescription, target, and disease	Comprehensive analysis for TCM biological sciences	http://www.megabionet.org/tcmid/	[21]
	TcmSP	Traditional Chinese medicine systems pharmacology database and analysis platform: providing information on relationships between drugs, targets, and diseases	Comprehensive analysis for TCM biological sciences	http://tcmspnw.com	[165]
	SIRC-TCM	Traditional Chinese medicine information database: providing information on formulae, medicinal herbs, and herbal ingredients	TCM formula and medical compound retrieval	http://www.tcm120.com/1w2k/tcm_species.asp	[166]

^#^B: biomolecular databases; D: disease/phenotype databases; C: chemical/drug-related databases; T: TCM related-databases.

**Table 2:** Network analysis tools.

Name/platform	Description	Type	Webpage
Cytoscape	An open source software platform for analyzing and visualizing complex networks: integrating a lot of plugins (Apps) concerning network analysis, communication scripting, and functional enrichment for biological network analysis. In addition, the package allows third-party developers to extend functionality of network analysis based on Java script [22, 23].	Free	http://www.cytoscape.org/

Pajek	A particularly useful package for the analysis of very large networks: integrating many network analysis methods. Thanks to its specific.net data file type, most of the algorithms of network analysis run quickly and scale well [167].	Free	http://pajek.imfm.si/doku.php

NetworkX	A Python-based package for comprehensive analysis of complex networks: integrating many network analysis methods including network structure and analysis measures.	Free	http://networkx.github.io/

Ucinet	A comprehensive package for the analysis of network: providing many network analysis methods as well as multivariate statistics. In addition, the package has strong matrix analysis such as matrix algebra and can be used to analyze different mode network data.	Commercial use	http://www.analytictech.com/ucinet/

NetMiner	An application software for exploratory analysis and visualization of large network data: providing 73 kinds of network analysis modules, 25 kinds of statistic and mining analysis modules, 28 kinds of visualization algorithms, 21 kinds of data transform modules.	Commercial use	http://www.netminer.com/

Guess	An exploratory data analysis and visualization tool for graphs and networks supporting Python which facilitate to the researcher working on graph structures in their own manners.	Free	http://graphexploration.cond.org/

Complex Networks Package for Matlab	Providing a comprehensive framework for both static and dynamic network analysis in Matlab.	Free	http://www.levmuchnik.net/Content/Networks/ComplexNetworksPackage.html

QuACN	An R Package for analyzing complex biological networks: providing function of analysis, classification and comparison for networks by different topological network descriptors [168, 169].	Free	http://cran.r-project.org/web/packages/QuACN/

**Table 3:** Network-based subgroup analysis approaches in TCM.

Algorithm	Description	Application and findings
BK	Bron-Kerbosch algorithm: an efficient algorithm for finding all maximal cliques of a network. The recursive procedure for optimizing candidate selection is performed based on the three different sets (R, P, X) of nodes, where R represents the currently growing clique (initially empty), P denotes prospective nodes, and X stands for the nodes already processed [69].	Applied for the discovery of basic formula (BF) in herbal prescriptions of the famous TCM expert. Three BFs for psoriasis and four BFs for eczema were found [58].

K-core	A subnetwork detecting methods to find the required clusters in which all the nodes have at least k degree [64].	Applied for the subnetworks analysis of TCM ingredients target-target network, as well as for the measuring centrality of nodes by “*K* value” [77].
		Applied for clustering symptoms for differentiating TCM syndrome of coronary heart disease based on the symptom-symptom network [76].

IPCA	A network-based clustering algorithm to identify subgroups based on the new topological structure [170].	Applied for clustering functional proteins of PPIs network based on TCM cold and hot syndromes [80] or TCM therapy [123].

CPM	Clique percolation Method for finding such a subgroup that corresponds to fully connected k nodes [56].	Applied for detecting synergistic or antagonistic subgroups of clinical factors networks in TCM tumor treatment [78].

SA	A simulated annealing algorithm, which is a generic probabilistic metaheuristic of the global optimizing for decomposing the networks [73].	Applied for subgroups detecting based on pathway-pathway association network for salvianolic acid B [79].

**Table 4:** Computational methods/algorithms for network pharmacology.

Type	Method and algorithm	Description	Application^#^
Network based	drugCIPHER	A network-based method for drug-target identification based on three linear regression models which integrates drug therapeutic similarity, chemical similarity, and the relevance of targets on PPIs network, respectively [91].	^ H^[91, 124, 132]
	DMIM	A distance-based mutual information model for indicating the relationship of herbs in TCM formulas [59].	^ H^[59]
	WNBI	A weight network-based inference method for drug-target prediction by integrating drug similarity and known target similarity [93].	^ H^[93]
	CIPHER	A computational framework based on a regression model which integrates PPIs, disease phenotype similarities, and gene-phenotype relationships [101].	^ D^[101]
	LMMA	A reliable approach for constructing disease-related gene network, which combines literature mining and microarray analysis [102].	^ D^[102]
	ClustEx	A two-step method based on module identification in PPIs network by integrating the time-course microarray data for specific disease-related gene discovery [171].	^ D^[171]
	MIClique	Identifying disease gene subsets by the combination of mutual information and clique analysis for biological networks [103].	^ D^[103]
	rcNet	A coupling ridge regression model established based on the known phenotype-gene network for predicting the unknown ones by maximizing the coherence between them [172].	^ D^[172]
	WSM	A similarity based method for weighted networks matching [104].	^ D^[104]
	SCAN	A structural clustering algorithm based on biological networks for functional modules discovery [173].	^ D^[173]
	CIPHER-HIT	A hitting-time-based method for predicting disease genes, which combined the modularity measure into the network inference [53].	^ I^[53]
	ComCIPHER	An efficient approach for identifying drug-gene-disease comodules underlying the gene closeness data [116].	^ I^[116]
	PPA	Ping-Pong algorithm: an efficient algorithm for predicting drug-gene associations based on multitypes of data [115].	^ I^[115]
	ISA	Iterative signature algorithm for searching the modules in heterogeneous network [118].	^ I^[118]
	NSS	A network stratification strategy to analyze conglomerate networks [174].	^ I^[174]

Machine learning/others	KNN	K nearest neighbor algorithm: a classical supervised classification algorithm based on closest training samples in the feature space.	^ H^[81]
	SVM	Support vector machine: a supervised kernel based classification algorithm based on the support vectors which are obtained after the training process by transforming original space into kernel space.	^ B^[82–84, 96, 97, 175]
	GIP	Gaussian Interaction profile: an efficient classification algorithm for predicting drug-target by constructing a kernel function from the known drug-target interaction profiles [176].	^ H^[176]
	RF	Random forest: an ensemble learning method for classification based on a multitude of trained decision trees.	^ B^[82, 83, 177]
	Bayesian classifiers	A popular supervised classification method based on probabilistic graphical model.	^ B^[85–87, 98, 99]
	SOM	Self-organizing maps: a unsupervised technology based on competition among the output neurons for assignment of the input vectors to map input observations to an output space represented by a grid of output neurons for similarity assessment.	^ B^[88, 89]
	SEM	Similarity ensemble methods: usually based on several similarity index such as Tanimoto coefficient(Tc) [107, 108] or Jaccard coefficient (Jc) [109].	^ B^[38, 110, 111]
	PCA	Principal component analysis: a classical data reduction technique for revealing the interrelationship among many variables by creating linear combinations of them into a few new variables to facilitate clustering and model analysis.	^ B^[100, 124, 178]

Application^#^: ^H^herb-related networks construction; ^D^disease-related networks construction; ^I^integrative analysis; ^B^both herb- and- disease-related networks construction.

**Table 5:** TCM network pharmacology for understanding the treatment principle of complex diseases.

Disease/action^#^	Related ingredient/herb/formula	Reference
T2DM	Tangminling pills	[134]
APL	Realgar-indigo naturalis formula	[179]
RA	Yishen juanbi tablet	[123]
	Qing-Luo-Yin	[124]
	Wu Tou Tang	[77]
CVD	Ligusticum Chuanxiong Hort., Dalbergia Odorifera T. Chen and Corydalis Yanhusuo WT Wang	[83]
	Radix Astragali Mongolici, Radix Puerariae Lobatae, Radix Ophiopogonis Japonici, and Radix Salviae Miltiorrhiza	[120]
	Compound Danshen formula	[37, 180]
	Astragaloside IV	[119]
	Salvianolic acid B	[79]
	Radix Curcumae formula	[111]
	Salvia Miltiorrhiza, Safflower, Ligustici Chuanxiong, Herba Erigerontis, Semen Persicae, Panax Notoginseng, Radix Paeoniae Rubra	[181]
	Tiao-Pi-Hu-Xin formula	[182]
OA	Chuanxiong Rhizome, Paeonia Albifora Pall	[113]
	Tao-Hong-Si-Wu decoction	[183]
Alzheimer	Ginkgo Biloba, Huperzia Serrata, Melissa Officinalis, Salvia Officinalis	[184]
Anti-angiogenesis	Sixty-one herbal ingredients	[38]
Sepsis	Xue-Bi-Jing formula	[185]
Cancer	Kang Ai Pian	[186]
	Ganoderic acid D	[187]
Influenza	Lonicera Japonica and Fructus Forsythiae	[188]
	Maxingshigan-Yinqiaosan formula	[189]
Hepatoprotection	Yin-Chen-Hao-Tang	[190]
GBS	Gui-Zhi-Fu-Ling capsule	[191]
AWI	Zhike Chuanbei Pipa dropping pills	[192]
CKD	Sixty-two herbs	[193]

^#^T2DM: type II diabetes mellitus; APL: acute promyelocytic leukemia; RA: rheumatoid arthritis; CVD: cardiovascular disease; OA: osteoarthritis; GBS: gynecological blood stasis; AWI: airway inflammation; CKD: chronic kidney disease.

## References

[1] Barnes PM, Bloom B, Nahin RL (2008). Complementary and alternative medicine use among adults and children: United States, 2007. *National Health Statistics Reports*.

[2] Schena M, Shalon D, Davis RW, Brown PO (1995). Quantitative monitoring of gene expression patterns with a complementary DNA microarray. *Science*.

[3] Yildirim MA, Goh K-I, Cusick ME, Barabási A-L, Vidal M (2007). Drug-target network. *Nature Biotechnology*.

[4] Paolini GV, Shapland RHB, Van Hoorn WP, Mason JS, Hopkins AL (2006). Global mapping of pharmacological space. *Nature Biotechnology*.

[5] Nacher JC, Schwartz J-M (2008). A global view of drug-therapy interactions. *BMC Pharmacology*.

[6] Gregori-Puigjané E, Mestres J (2008). A ligand-based approach to mining the chemogenomic space of drugs. *Combinatorial Chemistry & High Throughput Screening*.

[7] Pujol A, Mosca R, Farrés J, Aloy P (2010). Unveiling the role of network and systems biology in drug discovery. *Trends in Pharmacological Sciences*.

[8] Li S (2007). Framework and practice of network-based studies for Chinese herbal formula. *Journal of Chinese Integrative Medicine*.

[9] Li S (2011). Network target: a starting point for traditional Chinese medicine network pharmacology. *Zhongguo Zhongyao Zazhi*.

[10] Li S, Zhang B (2013). Traditional Chinese medicine network pharmacology: theory, methodology and application. *Chinese Journal of Natural Medicines*.

[11] Perini L (2005). Explanation in two dimensions: diagrams and biological explanation. *Biology & Philosophy*.

[12] Kanehisa M (2002). The KEGG database. *In Silico Simulation of Biological Processes*.

[13] Keshava Prasad TS, Goel R, Kandasamy K (2009). Human protein reference database—2009 update. *Nucleic Acids Research*.

[14] Berman HM, Westbrook J, Feng Z (2000). The protein data bank. *Nucleic Acids Research*.

[15] Zhu F, Han B, Kumar P (2010). Update of TTD: therapeutic target database. *Nucleic Acids Research*.

[16] Kamburov A, Stelzl U, Lehrach H (2013). The ConsensusPathDB interaction database: 2013 update. *Nucleic Acids Research*.

[17] Chen JY, Mamidipalli SR, Huan TX (2009). HAPPI: an online database of comprehensive human annotated and predicted protein interactions. *BMC Genomics*.

[18] McKusick-Nathans Institute of Genetic Medicine, Johns Hopkins University Online Mendelian Inheritance in Man, OMIM. http://omim.org/.

[19] Knox C, Law V, Jewison T (2011). DrugBank 3.0: a comprehensive resource for ‘Omics’ research on drugs. *Nucleic Acids Research*.

[20] Overington JP (2009). ChEMBL: large-scale mapping of medicinal chemistry and pharmacology data to genomes. *Abstracts of Papers of the American Chemical Society*.

[21] Xue RC, Fang Z, Zhang MX (2013). TCMID: traditional Chinese medicine integrative database for herb molecular mechanism analysis. *Nucleic Acids Research*.

[22] Shannon P, Markiel A, Ozier O (2003). Cytoscape: a software environment for integrated models of biomolecular interaction networks. *Genome Research*.

[23] Smoot ME, Ono K, Ruscheinski J, Wang P-L, Ideker T (2011). Cytoscape 2.8: new features for data integration and network visualization. *Bioinformatics*.

[24] Chatr-Aryamontri A, Breitkreutz BJ, Heinicke S (2013). The BioGRID interaction database: 2013 update. *Nucleic Acids Research*.

[25] Gao J, Ade AS, Tarcea VG (2009). Integrating and annotating the interactome using the MiMI plugin for cytoscape. *Bioinformatics*.

[26] Pentchev K, Ono K, Herwig R, Ideker T, Kamburov A (2010). Evidence mining and novelty assessment of protein-protein interactions with the consensusPathDB plugin for Cytoscape. *Bioinformatics*.

[27] Hernandez-Toro J, Prieto C, De Las Rivas J (2007). APID2NET: unified interactome graphic analyzer. *Bioinformatics*.

[28] Vailaya A, Bluvas P, Kincaid R, Kuchinsky A, Creech M, Adler A (2005). An architecture for biological information extraction and representation. *Bioinformatics*.

[29] Azmi AS (2012). Network pharmacology for cancer drug discovery: are we there yet?. *Future Medicinal Chemistry*.

[30] Feldman I, Rzhetsky A, Vitkup D (2008). Network properties of genes harboring inherited disease mutations. *Proceedings of the National Academy of Sciences of the United States of America*.

[31] Goh K-I, Cusick ME, Valle D, Childs B, Vidal M, Barabási A-L (2007). The human disease network. *Proceedings of the National Academy of Sciences of the United States of America*.

[32] Xu JZ, Li YJ (2006). Discovering disease-genes by topological features in human protein-protein interaction network. *Bioinformatics*.

[33] Gandhi TKB, Zhong J, Mathivanan S (2006). Analysis of the human protein interactome and comparison with yeast, worm and fly interaction datasets. *Nature Genetics*.

[34] He MM, Smith AS, Oslob JD (2005). Medicine: small-molecule inhibition of TNF-*α*. *Science*.

[35] Moerke NJ, Aktas H, Chen H (2007). Small-molecule inhibition of the interaction between the translation initiation factors eIF4E and eIF4G. *Cell*.

[36] Yu HY, Kim PM, Sprecher E, Trifonov V, Gerstein M (2007). The importance of bottlenecks in protein networks: correlation with gene essentiality and expression dynamics. *PLoS Computational Biology*.

[37] Li X, Xu X, Wang J (2012). A system-level investigation into the mechanisms of Chinese Traditional Medicine: Compound Danshen Formula for cardiovascular disease treatment. *PLoS One*.

[38] Li S, Zhang B, Zhang N (2011). Network target for screening synergistic drug combinations with application to traditional Chinese medicine. *BMC Systems Biology*.

[39] Odibat O, Reddy CK (2012). Ranking differential hubs in gene co-expression networks. *Journal of Bioinformatics and Computational Biology*.

[40] Sharan R, Ulitsky I, Shamir R (2007). Network-based prediction of protein function. *Molecular Systems Biology*.

[41] Kotlyar M, Fortney K, Jurisica I (2012). Network-based characterization of drug-regulated genes, drug targets, and toxicity. *Methods*.

[42] Köhler S, Bauer S, Horn D, Robinson PN (2008). Walking the interactome for prioritization of candidate disease genes. *American Journal of Human Genetics*.

[43] Li Y, Patra JC (2010). Genome-wide inferring gene-phenotype relationship by walking on the heterogeneous network. *Bioinformatics*.

[44] Pan RK, Chatterjee N, Sinha S (2010). Mesoscopic organization reveals the constraints governing Caenorhabditis elegans nervous system. *PLoS ONE*.

[45] Wang YY, Xu KJ, Song J (2012). Exploring drug combinations in genetic interaction network. *BMC Bioinformatics*.

[46] de Ruiter A, Oostenbrink C (2013). Protein-ligand binding from distancefield distances and hamiltonian replica exchange simulations. *Journal of Chemical Theory and Computation*.

[47] Sun JC, Zhao ZM (2010). A comparative study of cancer proteins in the human protein-protein interaction network. *BMC Genomics*.

[48] Choura M, Rebai A (2012). Topological features of cancer proteins in the human NR-RTK interaction network. *Journal of Receptors and Signal Transduction*.

[49] Hartsperger ML, Blöchl F, Stümpflen V, Theis FJ (2010). Structuring heterogeneous biological information using fuzzy clustering of k-partite graphs. *BMC Bioinformatics*.

[50] Liu X, Lu P, Zuo X (2012). Prediction of network drug target based on improved model of bipartite graph valuation. *Zhongguo Zhongyao Zazhi*.

[51] Shen CC, Liu Y (2012). A tripartite clustering analysis on microRNA, gene and disease model. *Journal of Bioinformatics and Computational Biology*.

[52] Heath LS, Sioson AA (2009). Semantics of multimodal network models. *IEEE/ACM Transactions on Computational Biology and Bioinformatics*.

[53] Yao X, Hao H, Li Y, Li S (2011). Modularity-based credible prediction of disease genes and detection of disease subtypes on the phenotype-gene heterogeneous network. *BMC Systems Biology*.

[54] Newman MEJ (2004). Detecting community structure in networks. *European Physical Journal B*.

[55] Du H, Feldman MW, Li S, Jin X (2007). An algorithm for detecting community structure of social networks based on prior knowledge and modularity. *Complexity*.

[56] Zhang SH, Ning XM, Zhang X-S (2006). Identification of functional modules in a PPI network by clique percolation clustering. *Computational Biology and Chemistry*.

[57] Jiang Y, Deng Z, Li R (2001). Basic formulae of Chinese materia medica and its meaning of clinics. *Study Journal of Traditional Chinese Medicine*.

[58] Yang M, Tian Y, Chen JL (2012). Application of bron-kerbosch algorithm for discovery of basic formulas of traditional Chinese medicine. *Zhongguo Zhong Yao Za Zhi*.

[59] Li S, Zhang B, Jiang D, Wei Y, Zhang N (2010). Herb network construction and co-module analysis for uncovering the combination rule of traditional Chinese herbal formulae. *BMC Bioinformatics*.

[60] Li S (2009). Network systems underlying traditional Chinese medicine syndrome and herb formula. *Current Bioinformatics*.

[61] Tu C-C, Cheng HJ (1998). Spectral methods for graph bisection problems. *Computers and Operations Research*.

[62] Larsson Träff J (2006). Direct graph k-partitioning with a Kernighan-Lin like heuristic. *Operations Research Letters*.

[63] Balasundaram B, Chandramouli SS, Trukhanov S (2010). Approximation algorithms for finding and partitioning unit-disk graphs into co-k-plexes. *Optimization Letters*.

[64] Batagelj V, Zaveršnik M (2011). Fast algorithms for determining (generalized) core groups in social networks. *Advances in Data Analysis and Classification*.

[65] Shi XH, LuoLiang L, Wan Y, Xu J (2006). A finding maximal clique algorithm for predicting loop of protein structure. *Applied Mathematics and Computation*.

[66] Gardiner EJ, Artymiuk PJ, Willett P (1997). Clique-detection algorithms for matching three-dimensional molecular structures. *Journal of Molecular Graphics and Modelling*.

[67] Artymiuk PJ, Spriggs RV, Willett P (2005). Graph theoretic methods for the analysis of structural relationships in biological macromolecules. *Journal of the American Society for Information Science and Technology*.

[68] Kose F, Weckwerth W, Linke T, Fiehn O (2001). Visualizing plant metabolomic correlation networks using clique-metabolite matrices. *Bioinformatics*.

[69] Bron C, Kerbosch J (1973). Algorithm 457: finding all cliques of an undirected graph. *Communications of the ACM*.

[70] He DX, Liu J, Yang B (2012). An ant-based algorithm with local optimization for community detection in large-scale networks. *Advances in Complex Systems*.

[71] Chandrasekharam R, Subhramanian S, Chaudhury S (1993). Genetic algorithm for node partitioning problem and applications in VLSI design. *IEE Proceedings E*.

[72] Liu DY, Jin D, Baquero C (2013). Genetic algorithm with a local search strategy for discovering communities in complex networks. *International Journal of Computational Intelligence Systems*.

[73] Hou J, Yan G-F, Fan Z (2011). Memoryless cooperative graph search based on the simulated annealing algorithm. *Chinese Physics B*.

[74] Jiang YW, Jia CY, Yu J (2013). An efficient community detection method based on rank centrality. *Physica A*.

[75] Ochab JK (2012). Maximal-entropy random walk unifies centrality measures. *Physical Review E*.

[76] Shi Q, Zhao H, Chen J (2012). Study on TCM syndrome identification modes of coronary heart disease based on data mining. *Evidence-Based Complementary and Alternative Medicine*.

[77] Yan QZ, Dan HW, Shu FT (2013). A systems biology-based investigation into the pharmacological mechanisms of Wu Tou Tang acting on rheumatoid arthritis by integrating network analysis. *Evidence-Based Complementary and Alternative Medicine*.

[78] Ming Y, Lijing J, Peiqi C (2012). Complex systems entropy network and its application in data mining for Chinese medicine tumor clinics. *World Science and Technology*.

[79] Ye L, He Y, Ye H (2012). Pathway-pathway network-based study of the therapeutic mechanisms by which salvianolic acid B regulates cardiovascular diseases. *Chinese Science Bulletin*.

[80] Chen G, Lu C, Zha Q (2012). A network-based analysis of traditional Chinese medicine cold and hot patterns in rheumatoid arthritis. *Complementary Therapies in Medicine*.

[81] He Z, Zhang J, Shi X-H (2010). Predicting drug-target interaction networks based on functional groups and biological features. *PLoS ONE*.

[82] Yu H, Chen JX, Xu X (2012). A systematic prediction of multiple drug-target interactions from chemical, genomic, and pharmacological data. *PLoS One*.

[83] Li B, Xu X, Wang X (2012). A systems biology approach to understanding the mechanisms of action of chinese herbs for treatment of cardiovascular disease. *International Journal of Molecular Sciences*.

[84] Cheng F, Zhou Y, Li J (2012). Prediction of chemical-protein interactions: multitarget-QSAR versus computational chemogenomic methods. *Molecular BioSystems*.

[85] Krueger BA, Weil T, Schneider G (2009). Comparative virtual screening and novelty detection for NMDA-GlycineB antagonists. *Journal of Computer-Aided Molecular Design*.

[86] Wang YH, Li Y, Ding J, Wang Y, Chang Y (2008). Prediction of binding affinity for estrogen receptor *α* modulators using statistical learning approaches. *Molecular Diversity*.

[87] Nigsch F, Bender A, Jenkins JL, Mitchell JBO (2008). Ligand-target prediction using winnow and naive bayesian algorithms and the implications of overall performance statistics. *Journal of Chemical Information and Modeling*.

[88] Bouvier G, Evrard-Todeschi N, Girault J-P, Bertho G (2010). Automatic clustering of docking poses in virtual screening process using self-organizing map. *Bioinformatics*.

[89] Schneider G, Tanrikulu Y, Schneider P (2009). Self-organizing molecular fingerprints: a ligand-based view on drug-like chemical space and off-target prediction. *Future Medicinal Chemistry*.

[90] Cleves AE, Jain AN (2006). Robust ligand-based modeling of the biological targets of known drugs. *Journal of Medicinal Chemistry*.

[91] Zhao S, Li S (2010). Network-based relating pharmacological and genomic spaces for drug target identification. *PLoS ONE*.

[92] Penrod NM, Cowper-Sal-Lari R, Moore JH (2011). Systems genetics for drug target discovery. *Trends in Pharmacological Sciences*.

[93] Cheng F, Zhou Y, Li W (2012). Prediction of chemical-protein interactions network with weighted network-based inference method. *PLoS One*.

[94] Cheng FX, Liu C, Jiang J (2012). Prediction of drug-target interactions and drug repositioning via network-based inference. *PLOS Computational Biology*.

[95] Zanzoni A, Soler-López M, Aloy P (2009). A network medicine approach to human disease. *FEBS Letters*.

[96] Li L, Chen HM, Liu C (2013). A robust hybrid approach based on estimation of distribution algorithm and support vector machine for hunting candidate disease genes. *The Scientific World Journal*.

[97] Zhu YN, Pan W, Shen XT (2009). Support vector machines with disease-gene-centric network penalty for high dimensional microarray data. *Statistics and Its Interface*.

[98] Denham MC, Whittaker JC (2003). A Bayesian approach to disease gene location using allelic association. *Biostatistics*.

[99] Zhang L, Mukherjee B, Ghosh M, Wu R (2006). Bayesian modeling for genetic association in case-control studies: accounting for unknown population substructure. *Statistical Modelling*.

[100] Podder S, Ghosh TC (2012). Evolutionary dynamics of human autoimmune disease genes and malfunctioned immunological genes. *BMC Evolutionary Biology*.

[101] Wu X, Jiang R, Zhang MQ (2008). Network-based global inference of human disease genes. *Molecular Systems Biology*.

[102] Li S, Wu L, Zhang Z (2006). Constructing biological networks through combined literature mining and microarray analysis: a LMMA approach. *Bioinformatics*.

[103] Zhang H, Song X, Wang H, Zhang X (2009). MIClique: an algorithm to identify differentially coexpressed disease gene subset from microarray data. *Journal of Biomedicine and Biotechnology*.

[104] Bhattacharjee A, Jamil HM (2012). WSM: a novel algorithm for subgraph matching in large weighted graphs. *Journal of Intelligent Information Systems*.

[105] Sun PG, Gao L, Han S (2011). Prediction of human disease-related gene clusters by clustering analysis. *International Journal of Biological Sciences*.

[106] Achenbach J, Tiikkainen P, Franke L, Proschak E (2011). Computational tools for polypharmacology and repurposing. *Future Medicinal Chemistry*.

[107] Mccormick WP, Lyons NI, Hutcheson K (1992). Distributional properties of Jaccard index of similarity. *Communications in Statistics-Theory and Methods*.

[108] Tanimoto T (1957). IBM internal report.

[109] Godden JW, Xue L, Bajorath J (2000). Combinatorial preferences affect molecular similarity/diversity calculations using binary fingerprints and Tanimoto coefficients. *Journal of Chemical Information and Computer Sciences*.

[110] Meng ZX, Shou DZ, Chao QC (2013). Predicting the drug safety for traditional Chinese medicine through a comparative analysis of withdrawn drugs using pharmacological network. *Evidence-Based Complementary and Alternative Medicine*.

[111] Tao W, Xu X, Wang X (2013). Network pharmacology-based prediction of the active ingredients and potential targets of Chinese herbal Radix Curcumae formula for application to cardiovascular disease. *Journal of Ethnopharmacology*.

[112] Jaccard index. http://en.wikipedia.org/wiki/Jaccard_index.

[113] Ye H-Z, Zheng C-S, Xu X-J, Wu M-X, Liu X-X (2011). Potential synergistic and multitarget effect of herbal pair Chuanxiong Rhizome-Paeonia Albifora Pall on osteoarthritis disease: a computational pharmacology approach. *Chinese Journal of Integrative Medicine*.

[114] Liao C-S, Lu K, Baym M, Singh R, Berger B (2009). IsoRankN: spectral methods for global alignment of multiple protein networks. *Bioinformatics*.

[115] Kutalik Z, Beckmann JS, Bergmann S (2008). A modular approach for integrative analysis of large-scale gene-expression and drug-response data. *Nature Biotechnology*.

[116] Zhao S, Li S (2012). A co-module approach for elucidating drug-disease associations and revealing their molecular basis. *Bioinformatics*.

[117] Li J, Lu C, Jiang M (2012). Traditional chinese medicine-based network pharmacology could lead to new multicompound drug discovery. *Evidence-Based Complementary and Alternative Medicine*.

[118] Bergmann S, Ihmels J, Barkai N (2003). Iterative signature algorithm for the analysis of large-scale gene expression data. *Physical Review E*.

[119] Zhao J, Yang P, Li F (2012). Therapeutic effects of astragaloside IV on myocardial injuries: multi-target identification and network analysis. *PLoS One*.

[120] Wang X, Xu X, Tao WY (2012). A systems biology approach to uncovering pharmacological synergy in herbal medicines with applications to cardiovascular disease. *Evidence-Based Complementary and Alternative Medicine*.

[121] Bohler C, Radner H, Ernst M (2012). Rheumatoid arthritis and falls: the influence of disease activity. *Rheumatology*.

[122] Liu J, Liu R-L (2011). The potential role of Chinese medicine in ameliorating extra-articular manifestations of rheumatoid arthritis. *Chinese Journal of Integrative Medicine*.

[123] Jiang M, Lu C, Chen G (2012). Understanding the molecular mechanism of interventions in treating rheumatoid arthritis patients with corresponding traditional chinese medicine patterns based on bioinformatics approach. *Evidence-Based Complementary and Alternative Medicine*.

[124] Zhang B, Wang X, Li S (2013). An integrative platform of TCM network pharmacology and its application on a herbal formula, Qing-Luo-Yin. *Evidence-Based Complementary and Alternative Medicine*.

[125] Kong D-X, Li X-J, Zhang H-Y (2009). Where is the hope for drug discovery? Let history tell the future. *Drug Discovery Today*.

[126] Xu X, Zhang WX, Huang C (2012). A novel chemometric method for the prediction of human oral bioavailability. *International Journal of Molecular Sciences*.

[127] Li S, Zhang ZQ, Wu LJ, Zhang XG, Li YD, Wang YY (2007). Understanding ZHENG in traditional Chinese medicine in the context of neuro-endocrine-immune network. *IET Systems Biology*.

[128] Ma T, Tan C, Zhang H, Wang M, Ding W, Li S (2010). Bridging the gap between traditional Chinese medicine and systems biology: the connection of Cold Syndrome and NEI network. *Molecular BioSystems*.

[129] Li R, Ma T, Gu J, Liang X, Li S (2013). Imbalanced network biomarkers for traditional Chinese medicine Syndrome in gastritis patients. *Scientific Reports*.

[130] Jiang B, Liang XJ, Chen Y (2012). Integrating next-generation sequencing and traditional tongue diagnosis to determine tongue coating microbiome. *Scientific Reports*.

[131] Lu C, Niu X, Xiao C (2012). Network-based gene expression biomarkers for cold and heat patterns of rheumatoid arthritis in traditional chinese medicine. *Evidence-Based Complementary and Alternative Medicine*.

[132] Zhang B, Liu L, Zhao S (2013). Vitexicarpin acts as a novel angiogenesis inhibitor and its target network. *Evidence-Based Complementary and Alternative Medicine*.

[133] Zhang A, Sun H, Yang B, Wang X (2012). Predicting new molecular targets for rhein using network pharmacology. *BMC Systems Biology*.

[134] Gu J, Zhang H, Chen L, Xu S, Yuan G, Xu X (2011). Drug-target network and polypharmacology studies of a Traditional Chinese Medicine for type II diabetes mellitus. *Computational Biology and Chemistry*.

[135] Haiyu X, Ye T, Peng L (2013). A computational drug-target network for Yuanhu Zhitong prescription. *Evidence-Based Complementary and Alternative Medicine*.

[136] Ming Y, Yu T, JiaLei C (2013). Application of complex systems entropy network for discovering traditional herb-pairs in traditional Chinese medicine prescriptions. *Pharmaceutical Care and Research*.

[137] Run SZ, Xue ZZ, Nai LY (2010). Study on compounding rules of Chinese herb prescriptions for treating syndrome of liver and spleen disharmony by scale-free network. *World Science and Technology*.

[138] Jie L, Hong SL, Wei H (2009). Primary study of characteristics of lung cancer TCM treatment by data ming. *World Science and Technology*.

[139] Brown KR, Jurisica I (2005). Online predicted human interaction database. *Bioinformatics*.

[140] Franceschini A, Szklarczyk D, Frankild S (2013). STRING v9. 1: protein-protein interaction networks, with increased coverage and integration. *Nucleic Acids Research*.

[141] Gao ZT, Li HL, Zhang HL (2008). PDTD: a web-accessible protein database for drug target identification. *BMC Bioinformatics*.

[142] Chan WM, Consortium U (2010). The UniProt Knowledgebase (UniProtKB): a freely accessible, comprehensive and expertly curated protein sequence database. *Genetics Research*.

[143] Whirl-Carrillo M, McDonagh EM, Hebert JM (2012). Pharmacogenomics knowledge for personalized medicine. *Clinical Pharmacology & Therapeutics*.

[144] Xenarios I, Salwínski Ł, Duan XJ, Higney P, Kim S-M, Eisenberg D (2002). DIP, the database of interacting proteins: a research tool for studying cellular networks of protein interactions. *Nucleic Acids Research*.

[145] Huang H, Wu XG, Pandey R (2012). C^2^Maps: a network pharmacology database with comprehensive disease-gene-drug connectivity relationships. *BMC Genomics*.

[146] Schuierer S, Tranchevent L-C, Dengler U, Moreau Y (2010). Large-scale benchmark of endeavour using MetaCore maps. *Bioinformatics*.

[147] Ekins S, Bugrim A, Brovold L (2006). Algorithms for network analysis in systems-ADME/Tox using the MetaCore and MetaDrug platforms. *Xenobiotica*.

[148] Nishimura D (2001). BioCarta. *Biotech Software & Internet Report*.

[149] Fazekas D, Koltai M, Turei D (2013). SignaLink 2-a signaling pathway resource with multi-layered regulatory networks. *BMC Systems Biology*.

[150] Vastrik I, D’Eustachio P, Schmidt E (2007). Reactome: a knowledge base of biologic pathways and processes. *Genome Biology*.

[151] Kandasamy K, Sujatha Mohan S, Raju R (2010). NetPath: a public resource of curated signal transduction pathways. *Genome Biology*.

[152] Forbes SA, Bindal N, Bamford S (2011). COSMIC: mining complete cancer genomes in the catalogue of somatic mutations in cancer. *Nucleic Acids Research*.

[153] Robinson PN, Köhler S, Bauer S, Seelow D, Horn D, Mundlos S (2008). The human phenotype ontology: a tool for annotating and analyzing human hereditary disease. *American Journal of Human Genetics*.

[154] Kuhn M, Szklarczyk D, Franceschini A (2012). STITCH 3: zooming in on protein-chemical interactions. *Nucleic Acids Research*.

[155] Masciocchi J, Frau G, Fanton M (2009). MMsINC: a large-scale chemoinformatics database. *Nucleic Acids Research*.

[156] ChemicalBook. http://www.chemicalbook.com/.

[157] Kjaerulff SK, Wich L, Kringelum J (2013). ChemProt-2. 0: visual navigation in a disease chemical biology database. *Nucleic Acids Research*.

[158] LookChem. http://www.lookchem.com/.

[159] Pence HE, Williams A (2010). Chemspider: an online chemical information resource. *Journal of Chemical Education*.

[160] Ye H, Ye L, Kang H (2011). HIT: linking herbal active ingredients to targets. *Nucleic Acids Research*.

[161] Fang X, Shao L, Zhang H, Wang S (2005). CHMIS-C: a comprehensive herbal medicine information system for cancer. *Journal of Medicinal Chemistry*.

[162] Chen CY-C (2011). TCM Database@Taiwan: the world’s largest traditional Chinese medicine database for drug screening In Silico. *PLoS ONE*.

[163] Fang Y-C, Huang H-C, Chen H-H, Juan H-F (2008). TCMGeneDIT: a database for associated traditional Chinese medicine, gene and disease information using text mining. *BMC Complementary and Alternative Medicine*.

[164] Traditional Chinese Medicine Information Database http://tcm.cz3.nus.edu.sg/group/tcm-id/tcmid_ns.asp.

[165] Lab of Systems Pharmacology for Chinese Traditional Medicine TcmSP:Traditonal Chinese Medicine Systems Pharmacology Database and Analysis Platform. http://tcmspnw.com/login_clearSession.

[166] Shanghai R&D Public Service Platform.

[167] Batagelj V, Mrvar A (2002). Pajek—analysis and visualization of large networks. *Graph Drawing*.

[168] Mueller LAJ, Kugler KG, Graber A, Emmert-Streib F, Dehmer M (2011). Structural measures for network biology using QuACN. *BMC Bioinformatics*.

[169] Mueller LAJ, Kugler KG, Dander A, Graber A, Dehmer M (2011). QuACN: an R package for analyzing complex biological networks quantitatively. *Bioinformatics*.

[170] Li M, Chen J-E, Wang J-X, Hu B, Chen G (2008). Modifying the DPClus algorithm for identifying protein complexes based on new topological structures. *BMC Bioinformatics*.

[171] Gu J, Chen Y, Li S, Li Y (2010). Identification of responsive gene modules by network-based gene clustering and extending: application to inflammation and angiogenesis. *BMC Systems Biology*.

[172] Hwang TH, Zhang W, Xie MQ, Liu J, Kuang R (2011). Inferring disease and gene set associations with rank coherence in networks. *Bioinformatics*.

[173] Ding YJ, Chen MJ, Liu ZC (2012). atBioNet—an integrated network analysis tool for genomics and biomarker discovery. *BMC Genomics*.

[174] Zhang M, Lu LJ (2010). Investigating the validity of current network analysis on static conglomerate networks by protein network stratification. *BMC Bioinformatics*.

[175] Huang L-C, Wu X, Chen JY (2011). Predicting adverse side effects of drugs. *BMC Genomics*.

[176] van Laarhoven T, Nabuurs SB, Marchiori E (2011). Gaussian interaction profile kernels for predicting drug-target interaction. *Bioinformatics*.

[177] Bureau A, Dupuis J, Falls K (2005). Identifying SNPs predictive of phenotype using random forests. *Genetic Epidemiology*.

[178] Strömbergsson H, Kleywegt GJ (2009). A chemogenomics view on protein-ligand spaces. *BMC Bioinformatics*.

[179] Zhao J, Jiang P, Zhang W (2010). Molecular networks for the study of TCM pharmacology. *Briefings in Bioinformatics*.

[180] Li X, Wu L, Fan X (2011). Network pharmacology study on major active compounds of Fufang Danshen formula. *Zhongguo Zhongyao Zazhi*.

[181] Ming FH, Yan LZ, Zhen ZR (2012). Study on mechanism of blood-activating and stasis-dissolving Herbs on Coronary heart disease in molecular level by network pharmacology. *World Science and Technology*.

[182] Yan FZ, Zhi WX, Hai QA (2012). Pharmacodynamic research of spleen- regulating and heart-nourishing formula based on network features. *Traditional Chinese Drug & Research Clinical Pharmacology*.

[183] Xian XL (2013). Network pharmacology-based prediction of the multi-target capabilities of the compounds in Taohong Siwu decoction, and their application in osteoarthritis. *Experimental and Therapeutic Medicine*.

[184] Sun Y, Zhu R, Ye H (2013). Towards a bioinformatics analysis of anti-Alzheimer's herbal medicines from a target network perspective. *Brief Bioinform*.

[185] Ma ST, Feng CT, Zhang XL (2013). The multi-target capabilities of the compounds in a TCM used to treat sepsis and their in silico pharmacology. *Complementary Therapies in Medicine*.

[186] Chan E, Tan M, Xin J, Sudarsanam S, Johnson DE (2010). Interactions between traditional Chinese medicines and Western therapeutics. *Current Opinion in Drug Discovery & Development*.

[187] Yue Q-X, Cao Z-W, Guan S-H (2008). Proteomics characterization of the cytotoxicity mechanism of ganoderic acid D and computer-automated estimation of the possible drug target network. *Molecular and Cellular Proteomics*.

[188] Wang X, Xu X, Li Y (2013). Systems pharmacology uncovers Janus functions of botanical drugs: activation of host defense system and inhibition of influenza virus replication. *Integrative Biology*.

[189] Dai W, Chen JX, Lu P (2013). Pathway Pattern-based prediction of active drug components and gene targets from H1N1 influenza's treatment with maxingshigan-yinqiaosan formula. *Molecular BioSystems*.

[190] Sun H, Zhang AH, Yan GL (2013). Proteomics study on the hepatoprotective effects of traditional Chinese medicine formulae Yin-Chen-Hao-Tang by a combination of two-dimensional polyacrylamide gel electrophoresis and matrix-assisted laser desorption/ionization-time of flight mass spectrometry. *Journal of Pharmaceutical and Biomedical Analysis*.

[191] Wei X, Liang C, Qiru F (2012). Action mechanism studies of GuizhiFuling Capsule: based on a bio-network analysis. *Computers and Applied Chemistry*.

[192] Yang H, Xing L, Zhou M-G (2012). Network pharmacological research of volatile oil from Zhike Chuanbei Pipa Dropping Pills in treatment of airway inflammation. *Chinese Traditional and Herbal Drugs*.

[193] Zhu W, Qiu XH, Xu XJ, Lu C (2010). Computational network pharmacological research of Chinese medicinal plants for chronic kidney disease. *Science China Chemistry*.

